# Determinants of Aortic Prosthesis Mismatch in a Brazilian Public Health System Hospital: Big Patients or Small Prosthesis?

**DOI:** 10.5935/abc.20190231

**Published:** 2020-01

**Authors:** Maria Estefania Otto, Fernando Antibas Atik, Marcelo do Nascimento Moreira, Luiz Carlos Madruga Ribeiro, Bianca Corrêa Rocha de Mello, Joyce Gomes Elias Lima, Maiara Sanchez Ribeiro, Ana Carolina Pereira Matos Domingues, Reyna Pinheiro Calzada, Armindo Jreige Jr., Larissa Lucas Schloicka, Philippe Pibarot

**Affiliations:** 1 Instituto de Cardiologia do Distrito Federal (ICDF), Brasília, DF - Brazil; 2 Québec Heart & Lung Institute - Valvular Heart Diseases, Quebec - Canada

**Keywords:** Heart Valve Prosthesis/surgery, Size Perception, Body Mass Index, Preoperative Care, Postoperative Care, Echocardiography/methods

## Abstract

**Background:**

Prosthesis-patient mismatch (PPM) is associated with worse outcomes.

**Objective:**

Determine the frequency and evaluate preoperatory variables independently associated with severe PPM in a tertiary hospital focused on Public Health Care.

**Methods:**

A total of 316 patients submitted to aortic valve replacement, who had echocardiography performed within the first 30 days after surgery, were retrospectively analyzed. The indexed effective orifice area (iEOA) of the prosthesis was used to classify the patients into three groups, according to PPM, considering body mass index (BMI): severe PPM (iEOA) < 0.65 cm^2^/m^2^), mild to moderate PPM (iEOA, 0.65 cm^2^/m^2^ - 0.85 cm^2^/m^2^) and without PPM (iEOA > 0.85 cm^2^/m^2^) for a BMI < 30 kg/m^2^ and severe PPM (iEOA) < 0.55 cm^2^/m^2^), mild to moderate (iEOA, 0.55 cm^2^/m^2^- 0.70 cm^2^/m^2^) and without PPM (iEOA > 0.7 cm^2^/m^2^) for a BMI > 30 kg/m^2^. Statistical significance was considered when p < 0.05.

**Results:**

iEOA was obtained in 176 patients. The frequency of severe and moderate PPM was 33.4% and 36.2%, respectively. Severe PPM patients were younger and had larger BMI, but smaller left ventricular outflow tract diameter (LVOTD). The independent variables used to predict severe PPM were male gender, BMI > 25 kg/m^2^, age < 60 years, LVOTD < 21 mm, and rheumatic etiology with an area under the ROC curve of 0.82.

**Conclusion:**

The frequency of severe PPM is high in a Brazilian population representative of the Public Health System, and it is possible to predict PPM from preoperative variables such as rheumatic valvular disease, gender, BMI, age and LVOTD.

## Introduction

The concept of prosthesis-patient mismatch (PPM) after aortic valve replacement (AVR) occurs when the indexed effective orifice area (iEOA) of the inserted prosthesis is too small in relation to patient body size.^1^ PPM was first described in 1978,^2^ and its negative impact on morbidity, mortality and left ventricular reverse remodeling has been established.^3-6^ Transprosthetic gradients in patients with PPM varies with cardiac output, which in turn is determined by body surface area (BSA), and the relation of iEOA and pressure gradient is curvilinear. Therefore, iEOA smaller than 0.85 cm^2^/m^2^ generates higher gradients with possible consequences to the left ventricle (LV).^2^

The incidence of PPM is variable and ranges from 20-70% for moderate and 5-20% for severe PPM.^2,3^ Severe PPM has been associated with a 1.8-fold increase in mortality.^3^ Many studies have reported an impact of PPM on early^7,8^ and late mortality,^5-8^ especially in patients with pre-existing LV dysfunction.^5,7^ PPM was also associated with reduced functional capacity, less regression of LV mass and accelerated bioprosthetic valve degeneration.^6^

Several factors were associated with the occurrence of severe PPM, including: advanced age,^3^ female gender,^4^ large body surface area (BSA) and body mass index (BMI), presence of diabetes, hypertension, small aortic valve annulus (< 21 mm),^5^ and bioprosthesis implantation.^6^

There are few studies on the incidence and impact of PPM in Brazil. Oliveira *et al.* observed that 17% of patients with EOA < 0.75 cm^2^/m^2^ showed no increased mortality during a 10-year follow-up.^9^ There are some interesting features specific to the Brazilian population, such as the higher prevalence of rheumatic fever, a large proportion of patients with a small BSA, and implantation of prosthesis with iEOAs not reported according to the normal reference values provided by the medical society guidelines and recommendations.^1,10^ Furthermore, the analysis of preoperative factors that predict the occurrence of PPM is essential for its prevention.^11,12^

The objective of this study was to assess the frequency of PPM in a representative population treated in the Brazilian Public Health System and to identify the preoperative factors that are associated with the occurrence of PPM.

## Methods

In this cross-sectional retrospective study, performed from January 2011 to July 2016, we included patients older than 18 years who underwent AVR. Patients who died prior the first postoperative echocardiography or with incomplete clinical and echocardiographic data were excluded. Informed consent was obtained from each patient and the study protocol conforms to the ethical guidelines of the 1975 Declaration of Helsinki as reflected in a priori approval by the institution’s human research committee.

All subjects underwent surgical AVR and transthoracic echocardiogram (ETT) within 30 days after surgery. Three hundred and sixteen (316) patients met the inclusion criteria. However, data of indexed EOA (iEOA) to determine the degree of PPM was available only in 176 patients. These data were not found in the echocardiogram report, nor were the values for calculation in images available at the hospital imaging server in 140 patients. After the publication of the European prosthetics guidelines^1^ there was mandatory standardization for calculation of iEOA in our Echocardiogram Laboratory.

The echocardiographic evaluation was performed following the recommendations of the American Society of Echocardiography Guideline, obtaining two-dimensional, pulsed and continuous Doppler and M mode images with Philips HDI 5000, HD 7, iE33 or GE E9 ultrasound systems with 2-5 Hz multifrequency transducer. The left atrial volume and ejection fraction (EF) were measured by Simpson's method (for LVEF < 53%) or Teicholz (for LVEF ≥ 53%). LV mass was obtained by the Devereux formula (measured from M or 2-dimensional mode) and indexed to the BSA.^13^ LV diameters were obtained by M or bi-dimensional mode.^13^ LV outflow tract (LVOT) was evaluated at the plane before the aortic valve,^1,10,14^ the peak and mean gradients, the velocity time integrals (VTI) ratio of the LVOT and aortic prosthesis and the calculation of EOA were performed according to the ASE recommendations. EOA = (LVOT area × LVOT VTI)/Aortic flow VTI).^1,10^ The calculation of EOA was indexed to BSA estimated by the Dubois and Dubois formula: BSA = (Weight^0.425^ × Height^0.725^) × 0.007184 and was used to identify the degree of PPM.^1,5^

### Definitions of PPM

Definition #1: PPM was defined as severe if iEOA was < 0.65 cm^2^/m^2^, moderate if iEAO was between 0.65 cm^2^/m^2^ and 0.85 cm^2^/m^2^ and absent if iEOA > 0.85 cm^2^/m^2^.

Definition #2: We also used the definition of PPM adjusted for high BMI as recommended by European recommendations.^1^ For BMI < 30 mg/kg, moderate PPD is considered if iEOA is < 0.70 cm^2^/m^2^ and severe if iEOA < 0.55 cm^2^/m^2^. Definition #3: Severe PPM was also defined on the basis of the mean transprosthetic gradient > 20 mmHg.

We tested three different definitions for PPM in this study population to check which of them would identify better variables associated with mismatch.

### Statistical analysis

Continuous variables with normal distribution were presented as mean and standard deviation and categorical variables in absolute numbers and percentages with confidence intervals, when necessary. Means of the three PPM groups were compared with one-way ANOVA after the Shapiro-Wilk normality test and Tukey's post hoc test. For categorical variables, a Chi-square test was use to compare proportions and frequencies. The association between preoperative variables and occurrence of severe PPM was assessed using the Poisson regression with robust variance model. In the univariate analysis, the association between each independent variable and the occurrence of PPM was assessed, and those that presented p < 0.1 were selected for entry into the multivariable analysis. The multivariable models were built by the consecutive exclusion of one variable from each complete model that presented the highest value of p of the Wald test, as described by Hosmer and Lemeshow. Data for multivariable models were complete for 148 patients.

A receiver operating curve (ROC) analysis was performed to assess the predictive value of the multivariable model for the prediction of severe PPM. ROC analysis was performed only for the PPM definition with more independent variables in the study, i.e., Definition #2.

The analyses were conducted using the SAS 9.4 software and p < 0.05 was considered significant.

## Results

### Frequency and Comparison of PPM Groups

Severe and moderate PPM occurred in 33.4% and 36.2% of patients, respectively. Tables 1 and 2 compare baseline clinical and echocardiographic characteristics of the 3 PPM groups. Even though 19% of the patients (34 patients out of 176 with PPD data) had rheumatic etiology, few presented significant mitral valve disease and underwent concomitant valve surgery (Table 1). There was loss of iEOA data in 140 patients with an average gradient of 18.7 ± 7 mmHg and a peak of 32.1 ± 5 mmHg. Patients with severe PPM were younger and had larger BSA and BMI, smaller LVOT diameter, and higher prevalence of rheumatic heart disease. There was low incidence of aortic root enlargement at the time of surgery in all groups. Table 3 shows the types and numbers of implanted prostheses and was presented in a descriptive way according to the type, number and category of PPD. There was a wide range of types and sizes of prostheses used in the AVR, which makes it impossible to analyze the association between prosthesis type, prosthesis number and degree of PPD. The data in Table 3 is too sparse to allow for any statistical model. Saint Jude bioprosthesis was implanted in 58% of patients (it is the most frequent), but cannot be tested as a determinant of PPM.

**Table 1:** Baseline clinical characteristics of the PPM groups (176 patients Definition #2)

	No PPM 30.4%(54)	Moderate PPM 36.2% (64)	Severe PPM 33.4% (58)	p
Age (years)	55 ± 17^┼^	60 ± 15^┼^*	52 ± 16*	0.0335
Female/male gender %	11/19	15/21.2	14/20.4	0.78
BSA (m^2^)	1.70 ± 0.24	1.71 ± 0.17*	1.8 ± 0.21*	0.016
BMI (kg/m^2^)	25 ± 3.37^┼^	26 ± 4.42^┼^*	27 ± 5.17*	0.03
SBP (mmHg)	120 ± 15^┼^	117 ± 18^┼^*	111 ± 15*	0.03
DBP (mmHg)	72 ± 14^┼^	66 ± 11^┼^	68 ± 13	0.028
HR bpm	83 ± 14	83 ± 14	87 ± 13	0.26
Hypertension%	17.6	22.1	17.6	0.54
Diabetes%	2.8	5.1	4.6	0.77
CABG%	6.3	8	4	0.25
Renal Disease	1	2	1	0.4
Aortic Root enlargement %	1.7	1.1	3.4	0.27
Mitral valve surgery %	0	1.14	0.57	0.63
Valve disease Etiology (%)				0.003
Rheumatic	4(9.5)	8(14.55)^┼^	22(43.1)^┼^	
Degenerative	21(50)	29(52.8)	19(37.3)	
Congenital (Bicuspid)	11(26.2)	10(8.2)	7(13.7)	
Aortic Root Dilation	6(14.3)	8(14.6)	3(5.9)	
Type of Prosthesis Biop./Mech. %	24/6.4	31/5.2	31.4/2	0.27

┼ p < 0.05 between no PPM and moderate PPM.

* p < 0.05 between moderate and severe PPM.

BSA: body surface area; BMI: body mass index; SBP: systolic blood pressure; DBP: diastolic blood pressure; HR: heart rate; CABG: coronary bypass graft; Biop.: biological; Mech: mechanical.

**Table 2:** Postoperative Doppler-Echocardiographic Data According to PPM Groups (176 patients- Definition# 2)

	No PPM 30.4% (54)	Moderate PPM 36.2% (64)	Severe PPM 33.4% (58)	p
LVEF %	57 ± 14%	60 ± 14%	58 ± 14%	0.75
Vmax Ao cm/s	273 ± 15	306 ± 25	335 ± 18	< 0.002
Peak Gradient (mmHg)	30 ± 14^┼^	37 ± 14^┼^*	45.1 ± 20*	< 0.0001
Mean Gradient (mmHg)	18 ± 8^┼^	21 ± 8^┼^*	28 ± 13*	<0.0001
EOA cm^2^	1.78 ± 0.43^┼^	1.3 ± 0.2^┼^*	0.52 ± 0.1*	< 0.0001
EOA/BSA cm^2^/m^2^	1.05 ± 0.17^┼^	0.73 ± 0.06^┼^*	0.51 ± 0.1*	< 0.0001
VTI LVOT/VTI Ao valve	0.49 ± 0.1^┼^	0.41 ± 0.07^┼^*	0.33 ± 0.08*	< 0.0001
LVOT diameter (cm)	2.15 ± 0.3^┼^	2.02 ± 0.24^┼^*	1.92 ± 0.22*	0.04
LV mass index g/m^2^	115 ± 42	119 ± 38	117 ± 35	0.84
LA index volume ml/m^2^	32 ± 12	33 ± 10	33 ± 12	0.72
Ascending Aorta cm	3.6 ± 0.72	3.6 ± 0.74	3.5 ± 0.56	0.29

┼ p < 0.05 between no PPM and moderate PPM.

* p < 0.05 between moderate and severe PPM.

LVEF: left ventricle ejection fraction; EAO: effective orifice area; BSA: body surface area; VTI: velocity time integral; LVOT: left ventricle outflow tract; Ao: aortic; LV: left ventricle; LA: left atrium. NA: not available.

**Table 3:** Type and number of implanted prosthesis

Types of Prostheses	Number	No PPM	Moderate PPM	Severe PPM
Labcor Biological	19	0	1	0
	25	5	1	0
Carpentier Edwards	19	0	0	1
	21	1	3	0
	23	3	1	2
	25	0	2	0
St Jude Biological	18	0	1	0
	19	1	1	1
	21	5	8	12
	23	8	20	21
	25	5	7	3
	27	6	1	3
St. Jude Mechanical	19	2	1	1
	21	0	3	1
	23	2	2	1
	25	1	2	1
	27	1	0	1
Non-Specific Mechanical	18	0	0	1
	19	0	0	1
	23	0	1	1
	25	2	0	0
	27	0	1	0
	28	0	1	0
Non-Specific Biological	19	0	1	0
	21	0	3	2
	23	6	2	1
	25	1	0	1
	27	0	1	1
Hancock Biological	23	0	0	2
	25	0	0	1
	27	0	0	1
Biocor Biological	23	1	0	0
Medtronic Mechanical	21	1	0	0

Descriptive Table of aortic prosthesis implanted in the present study.

* No description of prosthesis type in files or surgery report.

### Preoperative Determinants of Severe PPM

#### Determinants of severe PPM according to Definition #1 (indexed EOA< 0.65 cm^2^/m^2^)

In univariate analysis (Table 4), there was an association between severe PPM and the following variables: age < 60 years, BSA > 1.74 m^2^, rheumatic heart disease as the etiology of aortic valve disease and not performing aortic root enlargement. Multivariable analysis (Table 4) revealed that preoperative variables independently were the same as in univariate analysis, except for not performing aortic root enlargement. The tolerance indicator for multicollinearity was 0.78, indicating that there is no strong multicollinearity among the independent variables.

**Table 4:** Poisson Multivariable analysis for severe PPM (Definition #1; EOAi < 0.65 cm^2^/m^2^; n = 148 patients)

Variables	Crude RP		Adjusted RP	
	RP (CI 95 %)	p-value	RP (CI 95%)	p-value
**Gender**		**0.7982**		**-**
Female	1	-	-	-
Male	1.06 (0.67-1.67)	0.7982	-	-
**Age**		**0.0078**		**0.0134**
< 60 years	1.98 (1.20- 3.29)	0.0078	2.06 (1.16- 3.67)	
≥ 60 years	1	-	1	-
**BSA**		**0.0571**		**0.0176**
≤ 1.74 m^2^	1	-	1	-
> 1.74 m^2^	1.56 (0.99- 2.46)	0.0571	1.65 (1.09; 2.50)	
**BMI**		**0.0168**		**0.0030**
< 25 Kg/m^2^	1	-	1	-
> 25 Kg/m^2^	1.80 (1.11- 2.91)	0.0168	1.89 (1.24-2.87)	
**Main Diagnosis**		**0.6092**		**-**
Stenosis	1.28 (0.78- 2.12)	0.3283	-	-
Regurgitation	1	-	-	-
Balanced	1.11 (0.48- 2.55)	0.8039	-	-
**Etiology of AV Disease**		**0.0009**		**0.0028**
Rheumatic	3.50 (1.21-10.11)	0.0206	4.00 (1.49-10.77)	0.0060
Degenerative	1.56 (0.52- 4.67)	0.4262	2.17 (0.79-5.97)	0.1331
Congenital (bicuspid)	1.82 (0.57- 5.81)	0.3107	1.78 (0.63- 5.01)	0.2779
Aortic Root Dilation	1	-	1	-
**Reoperation**		**0.3379**		**-**
No	1	-	-	-
Yes	1.29 (0.76-2.19)	0.3379	-	-
**Hypertension**		**0.1400**		**-**
No	1.39 (0.90- 2.14)	0.1400	-	-
Yes	1	-	-	-
**Diabetes**		**0.4760**		**-**
No	1	-	-	-
Yes	1.23 (0.69- 2.20)	0.4760	-	-
**CABG**		**0.1013**		**-**
No	1.87 (0.88- 3.95)	0.1013	-	-
Yes	1	-	-	-
**Type of Prosthesis**		**0.1398**		**-**
Biop.	1.98 (0.80- 4.93)	0.1398	-	-
Mech	1	-	-	-
**LVOT Enlargement**		**0.0374**		**-**
No	1.86 (1.04- 3.34)	0.0374	-	-
Yes	1	-	-	-
**LV mass index**		**0.0902**		**-**
≤ 127 g/m^2^	1.48 (0.94- 2.32)	0.0902	-	-
> 127 g/m^2^	1	-	-	-
**Ejection Fraction**		**0.1093**		**-**
≤ 64 %	1.44 (0.92-2.25)	0.1093	-	-
> 64 %	1	-	-	-
**LVOT diameter**		**0.0069**		**< 0.0001**
≤ 2.1 cm	2.15 (1.23-3.74)	0.0069	2.88 (1.71-4.84)	
> 2.1 cm	1	-	1	-

EOAi: Effective orifice area index; RP: relative prevalence; CI: confidence interval; BSA: Body Surface Area; BMI: Body Mass Index; CABG: Coronary Artery Bypass Graft; LVOT: Left Ventricle Outflow Tract; LV: Left Ventricle; AV: aortic valve.

#### Determinants of severe PPM according to Definition #2 (indexed EOA < 0,65 cm^2^/m^2^ for patients with BMI < 30 kg/m^2^ and EOA < 0.55 cm^2^/m^2^ for BMI > 30 kg/m^2^

In addition to the independent variables described in the analysis above using the cut-off value of < 0.65 cm^2^/m^2^ for severe PPM, we found that male gender is an independent determinant of PPM when BMI is considered as a parameter for reclassification of severe PPM to iEOA ≤ 0.55 cm^2^/m^2^. However, BSA was not an independent variable within this new model. Univariate and multivariate analysis are shown in Table 5.

**Table 5:** Poisson Multivariable analysis for severe PPM (Definition #2: indexed EOA < 0.65 cm^2^/m^2^ for BMI < 30 kg/m^2^ and < 0.55 cm^2^/m^2^ for BMI ≥ 30 kg/m^2^; n = 148 patients)

Variables	Crude RP		Adjusted RP	
	RP (CI 95 %)	p-value	RP (CI 95%)	p-value
**Gender**		**0.6856**		**0,0255**
Female	1	-	-	-
Male	1.10 (0.68-1.79)	0.6856	1,67 (1,06-2,61)	
**Age**		**0.0057**		**0.0025**
< 60 years	2.17 (1,25- 3.75)	0.0057	2.6 (1.4- 4.84)	
≥ 60 years	1	-	1	-
**BSA**		**0.2015**	**-**	
≤ 1.74 m^2^	1	-	-	-
> 1.74 m^2^	1.36 (0.85- 2.19)	0.2015		-
**BMI**		**0.0657**		**0.0034**
< 25 Kg/m^2^	1	-	1	-
> 25 Kg/m^2^	1.59 (0.97- 2.61)	0.0657	1.95 (1.25-3.06)	
**Main Diagnosis**		**0.7166**		**-**
Stenosis	1.25 (0.73- 2.13)	0.4152	-	-
Regurgitation	1	-	-	-
Balanced	1.19 (0.51- 2.76)	0.6850	-	-
**Etiology of Aortic Valve Disease**		**0.0010**		**0.0030**
Rheumatic	3.33 (1.15-9.67)	0.0267	3,29 (1.22-8.92)	0.0190
Degenerative	1.56 (0.52- 4.67)	0.5545	1.95 (0.69-5.50)	0.2079
Congenital (bicuspid)	1.82 (0.57- 5.81)	0.4244	1.32 (0.44- 3.98)	0.6245
Aortic Root Dilation	1	-	1	-
**Reoperation**		**0.1909**		**-**
No	1	-	-	-
Yes	1.43 (0.84-2.44)	0.1909	-	-
**Hypertension**		**0.0820**		**-**
No	1.51 (0.95- 2.39)	0.0820	-	-
Yes	1	-	-	-
**Diabetes**		**0.9325**		**-**
No	1	-	-	-
Yes	0.97 (0.48- 1.97)	0.9325	-	-
**CABG**		**0.0815**		**-**
No	2.1 (0.91- 4.82)	0.0815	-	-
Yes	1	-	-	-
**Type of Prosthesis**		**0.1993**		**-**
Bio	1.82 (0.73- 4.53)	0.1993	-	-
Mec	1	-	-	-
**LVOT Enlargement**		**0.0186**		**-**
No	2.03 (1.13- 3.68)	0.0186	-	-
Yes	1	-	-	-
**LV mass index**		**0.2952**		**-**
≤ 127 g/m^2^	1.29 (0.80; 2.06)	0.2952	-	-
> 127 g/m^2^	1	-	-	-
**Ejection Fraction**		**0.0517**		**-**
≤ 64 %	1.61 (1.00; 2.60)	0.0517	-	-
> 64 %	1	-	-	-
**LVOT diameter**		**0.0042**		**< 0.0001**
≤ 2,1 cm	2.45 (1.33-4.52)	0.0042	3.58 (2.01-6.39)	
> 2,1 cm	1	-	1	-

EOAi: Effective orifice area index; RP: relative prevalence; CI: confidence interval; BSA: Body Surface Area; BMI: Body Mass Index; CABG: Coronary Artery Bypass Graft; LVOT: Left Ventricle Outflow Tract; LV: Left Ventricle; AV: aortic valve.

#### Determinants of severe PPM according to Definition #3 (mean prosthesis gradient ≥ 20 mmHg and iEOA ≤ 0,65 cm^2^/m^2^)

With this definition, only age < 60 years (PR: 3.33; IC 95%: 1.56-7.12) and LVOT diameter < 2.1 cm (PR = 1.68; IC 95%: 0.87-3.21) were independently associated with severe PPM. Complete analysis is described in Table 6.

**Table 6:** Poisson Multivariable analysis for severe PPM (Definition #3: mean transprosthetic gradient > 20 mmHg ; n = 148 patients)

Variables	Crude RP		Adjusted RP	
	RP (CI 95 %)	p-value	RP (CI 95%)	p-value
**Gender**		**0.5995**		**-**
Female	1	-	-	-
Male	1.17 (0.65-2.13)	0.5995	-	0.0004
**Age**		**0.0019**		**0.0004**
< 60 years	3.33 (1.56- 7.12)	0.0019	3.94(1.85- 8.39)	
≥ 60 years	1	-	1	-
**BSA**		**0.7720**	**-**	
≤ 1.74 m^2^	1	-	-	
> 1.74 m^2^	1.09 (0.62- 1.92)	0.7720		
**BMI**		**0.2905**		**-**
< 25 Kg/m^2^	1	-	1	-
> 25 Kg/m^2^	1.37 (0.76-2.47)	0.2905	-	-
**Main Diagnosis**		**0.4620**		**-**
Stenosis	1.54 (0.77- 3.06)	0.2178	-	-
Regurgitation	1	-	-	-
Balanced	1.48 (0.53- 4.14)	0.4531	-	-
**Etiology of Aortic Valve Disease**		**0.0035**		
Rheumatic	4.00 (1.04-15.43)	0.0441		
Degenerative	1.35 (0.33- 5.55)	0.6728		
Congenital (bicuspid)	2.12 (0.5- 9.07)	0.3087		
Aortic Root Dilation	1	-	-	-
**Reoperation**		**0.4442**		**-**
No	1	-	-	-
Yes	1.31 (0.65-2.63)	0.4442	-	-
**Hypertension**		**0.1297**		**-**
No	1.55 (0.88- 2.73)	0.1297	-	-
Yes	1	-	-	-
**DM**		**0.7270**		**-**
No	1	-	-	-
Yes	0.85 (0.34- 2.13)	0.7270	-	-
**CABG**		**0.1715**		**-**
No	1.95 (0.75- 5.08)	0.1715	-	-
Yes	1	-	-	-
**Type of Prosthesis**		**0.5560**		**-**
Bio	1.32 (0.52- 3.35)	0.5560	-	-
Mec	1	-	-	-
**LVOT Enlargement**		**0.0427**		**-**
No	2.19 (1.03- 4.66)	0.0427	-	-
Yes	1	-	-	-
**LV mass index**		**0.7019**		**-**
≤ 127 g/m^2^	1.12 (0.63-1.97)	0.7019	-	-
> 127 g/m^2^	1	-	-	-
**Ejection Fraction**		**0.0409**		**-**
≤ 64%	1.87 (1.03-3.4)	0.0409	-	-
> 64%	1	-	-	-
**LVOT diameter**		**0.1198**		**0.0138**
≤ 2,1 cm	1.68 (0.87-3.21)	0.1198	2.2 (1.17-4.14)	0.0138
> 2,1 cm	1	-	1	-

Legend: BSA: Body Surface Area; BMI: Body Mass Index; CABG: Coronary Artery Bypass Graft; LVOT: Left Ventricle Outflow Tract; LV: Left Ventricle.

#### Accuracy and Mathematical model for Prediction of Severe PPM with preoperative variables

**Figure 1:**
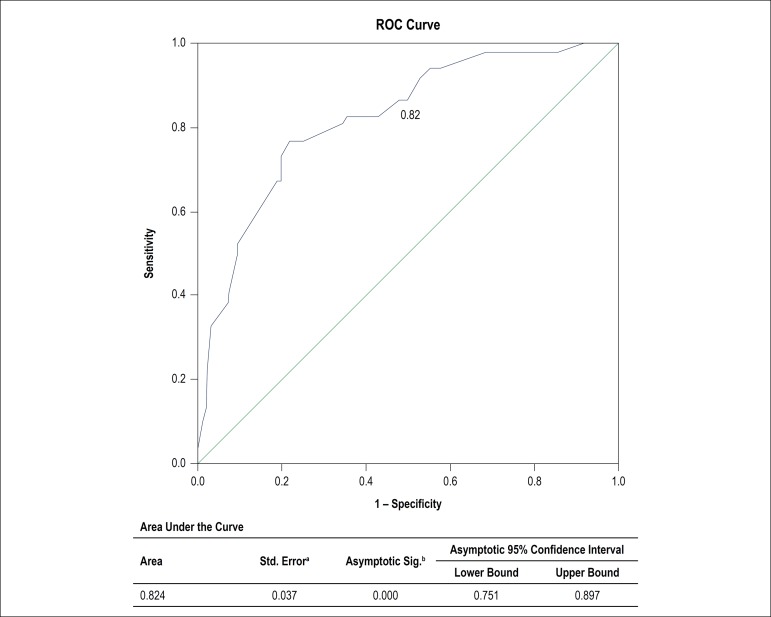
ROC curve: accuracy of the multivariable model for prediction of severe PPM (Definition #2:)

We tested the accuracy of the predictive model for severe PPM using *Definition # 2,* for its precision in identifying more independent variables compared with the other definitions. The area under the ROC curve was 0.82 (Figure 1).

In addition, to calculate the individual risk of a patient to develop severe PPM, we built a mathematical model summarized by a formula based on multivariate logistic regression analysis (Table 7). With this formula, it is possible to calculate the individual risk of PPM for each patient before surgery (Table 7).

**Table 7:** Formula for individual risk and coefficient of each variable for severe mismatch probability calculation (Definition #2)

Parameter		Estimate
Intercept		-5,54
Age	< 60	1,75
Male gender		0,79
LVOT Diameter	≤ 2.1	2,25
BMI	≥ 25 Kg/m^2^	1,12
Etiology of Aortic Valve Disease	Congenital (bicuspid)	0,41
Etiology of Aortic Valve Disease	Degenerative	1,10
Etiology of Aortic Valve Disease	Rheumatic	2,15
$\mathrm{Probability}\;\mathrm{of}\;\mathrm{severe}\;\mathrm{PPM}=\frac{1}{1+e^{\left(-5.54\;+1.75\;\mathrm{age}\;+\;0.79\;\mathrm{male}\;+\;2.25\;\mathrm{LVOT}\;\mathrm{diam}\;+\;1.12\;\mathrm{BMI}\;+\;0.41\;\mathrm{Etiol}\;\mathrm{Cong}\;+\;1.1\;\mathrm{Etiol}\;\deg\;+\;2.15\;\mathrm{Etiol}\;\mathrm{Rheum}\right)}}$		

Legend: PPM patient prosthesis mismatch; BMI: Body Mass Index; LVOT Diam: Left Ventricle Outflow Tract Diameter; Etiol Cong: Etiology Congenital; Etiol Deg: etiology degenerative; Etiol Rheum: Etiology Rheumatic. Observation: To determine the probabilities based on the above equation, one must use the design matrix on Table 6, if the variable present. For example: if a patient is < 60 years old, one must replace the variable age by the value 1 and multiply it by the value of its coefficient. If it is older than or equal to 60, one should replace the variable age by zero.

## Discussion

One of the main findings of this study is that frequency of severe PPM is high after AVR in patients treated in the Brazilian Public Health System in a representative tertiary center. The prevalence of severe PPM in this study was 33% compared to up to 20% previously described.^6,15^ Oliveira et al. described lower prevalence of PPM in Brazilian patients with small aortic annulus (16.8%). However, their cutoff points for definition of PPM was different from the present study.^9^ Another significant finding is that degenerative aortic stenosis is the main cause of aortic valve disease in our study (50%), but rheumatic etiology remains high, compared to data reported in developed countries (19%).^5,7^ In addition, rheumatic etiology is independently associated with the risk of severe PPM.

### PPM Characteristics

Similarly to other studies,^15,16^ patients with severe PPM had larger BSA and BMI ^15^ and smaller LVOT diameters.^6,15^ Patients with severe PPM in our study were younger compared to those in previous studies and mostly males.^5,7,8,15^ This finding could be explained by the inclusion of aortic regurgitation in our study, to explain male gender as an independent variable, and by the significant proportion of patients with rheumatic etiology, to explain the predominance of younger individuals with PPM.^18,19^

### Determinants of Severe PPM

A very important application of our findings is in the identification of independent preoperative variables which determine the risk of severe PPM. From these variables we built a predictive model that enables the identification of individual risk for development of severe PPM. This model can be used to identify patients at high risk for severe PPM prior to AVR and to implement preventive strategies.^18,19^

A larger BMI (> 25 kg/m^2^), male gender, smaller LVOT diameter (< 2.1 cm), younger age (≤ 60 years) and rheumatic etiology were determinants of high risk for severe PPM. Based on the predictive model proposed in this study, preventive strategies should be contemplated, including aortic root enlargement and implantation of prosthetic valves with superior hemodynamic performance with surgical or transcatheter procedure^11,15,18,19^ In this study population, transcatheter implantation is controversial because the procedure is approved for high surgical risk in patients, who are usually older and at a higher level of frailty. This study also raises the importance for improving the hemodynamic performance of the prosthetic valves implanted in the Brazilian Public Health System. However, we must consider the costs of using stentless prostheses in the public health system, which may have a negative impact cost to treat the population more comprehensively.

### Potential Limitations and Strengths of the Study

This was a retrospective study with limited data available of the iEOA in part of the population. It is important to emphasize that it was possible to obtain the indexed effective orifice data - the main parameter for the differentiation of PPM - in only 55% of the study population. Hence, the 45% of patients with missing data could generate bias and increase the prevalence of severe PPM. The type of prosthesis used was not found in some patients, in spite of being exhaustively searched in medical records. No long-term echocardiographic and clinical follow-up data was available to assess the effect of PPM on outcomes. However, this is the first study to show a high frequency of PPM in AVR performed in the Brazilian Public Health System. In addition, our study was able to build a mathematical model to predict PPM and find preoperative independent variables related to the implantation of small prosthesis. Further studies are needed to apply and validate this model in other populations.

## Conclusion

Severe aortic PPM is frequent among patients operated in the Brazilian Public Health System. The independent preoperative determinants of severe PPM in this population were: larger BMI, male gender, smaller LVOT diameter, younger age and rheumatic etiology. We developed a mathematical model including these preoperative variables in order to predict the risk of severe PPM prior to surgery. This model may be useful to implement prospective preventive strategies in patients identified as being at risk for severe PPM. Small prosthesis in big patients should be avoided.
